# Factors associated with long acting and permanent contraceptive methods use in Ethiopia

**DOI:** 10.1186/s40834-019-0091-3

**Published:** 2019-08-01

**Authors:** Gedefaw Abeje Fekadu, Akinyinka O. Omigbodun, Olumuyiwa A. Roberts, Alemayehu Worku Yalew

**Affiliations:** 1 Pan African University, Institute of Life and Earth Sciences (including health and Agriculture), University of Ibadan, Ibadan, Nigeria; 2 College of Medicine and Health Sciences, School of Public Health, Bahir Dar University, Bahir Dar, Ethiopia; 3 University College Hospital, University of Ibadan, Ibadan, Nigeria; 4 School of Public health, College of Medicine, Addis Ababa University, Addis Ababa, Ethiopia

**Keywords:** Ethiopia, Long acting and permanent contraceptives, Demographic and health survey

## Abstract

**Background:**

Long acting and permanent contraceptives methods are more effective, save cost and enable women to control their reproductive lives better. Although the Ethiopian government is promoting its use through various mechanisms, the level of use is low. Therefore, this study was designed to identify factors associated with long acting and permanent contraceptive methods use in Ethiopia.

**Methods:**

Four Ethiopian demographic and health survey data were used to examine trends of long acting and permanent contraceptive methods use. To identify factors associated with long acting and permanent contraceptive methods use, the 2016 Ethiopian demographic and health survey data was used. The data was accessed from the demographic and health survey program data base. Data analysis was done using Stata 15.1. Descriptive analysis was used to describe socio-economic and other variables of the study participants. Data were weighted and design effect was considered during analysis. Multicollinearity was assessed using variance inflation factor. Finally, multinomial logistic regression model was used to identify factors associated with long acting and permanent contraceptive methods use.

**Results:**

Long acting and permanent contraceptive methods use increased significantly from 0.6% in 2000 to 11.6% in 2016. The odds of long acting and permanent contraceptive methods use was higher among richer women (AOR 2.6; 95%CI 1.2–5.4), women who were sales workers (AOR 2.1; 95%CI 1.1–3.9) and women whose ideal number of children was high (AOR; 4.2, 95%CI 1.4–13.0). But the odds of long acting and permanent contraceptive methods use was lower among female headed households (AOR 0.2: 95%CI 0.1–0.5) and women who had history of abortion (AOR 0.2: 95%CI 0.1–0.5).

**Conclusion:**

Long acting and permanent contraceptive methods use increased significantly in Ethiopia. Wealth index, women’s occupation, ideal number of children, sex of head of the household and history of abortion were factors associated with long acting and permanent contraceptive methods use in Ethiopia. Improving economic status of women may help improve long acting and permanent contraceptive methods use in Ethiopia.

## Background

About 50% of women in developing regions of the world want to avoid pregnancy but only three quarters could do so. This causes unintended pregnancies [1]. Births from unintended pregnancies are more likely to suffer from many conditions [2]. In addition, unintended pregnancy increases unnecessary burden on public spending [3].

Long acting and permanent methods (LAPMs) are better options to reduce unintended pregnancies because these methods are more effective, save cost and enable women to control their reproductive lives better [4–6]. Use of long acting and permanent contraceptive methods can significantly increase contraceptive prevalence rate in countries with low contraceptive coverage [7]. Women using short acting contraceptives are 21 times more likely to have unintended pregnancy than women using long acting reversible and permanent methods [8]. A projection in SSA countries indicated that more than1.8 million unintended pregnancies would had been averted within 5 yrs period if 20% of women using oral contraceptives and injectable shift to implant [9].

On 2015, long acting and permanent contraceptive methods (Intra uterine device (IUD), implants and sterilization) accounted for 56% of contraceptive use globally. Nineteen percent of married or in-union women relied on female sterilization and 14% used IUD. Yet most contraceptive users in Africa depend on short term methods [7].

Lack of knowledge, myths, misconceptions and negative attitude about long acting and permanent contraceptive methods are the main barriers for long acting and permanent contraceptive method use in Ethiopia [10]. A study done at Adigrat town, Northwest Ethiopia reported that participants’ knowledge of long acting and permanent contraceptive methods was limited to recognizing the names of the methods. The study added that women had fears and rumors about these methods and prefer methods which do not require procedure [11]. Another study conducted in Dangila town, Ethiopia showed that men had low knowledge about vasectomy [12].

Ethiopia’s health service is structured into three-tier system: primary, secondary and tertiary. The primary level of care includes primary hospitals, health centers (HCs) and health posts (HPs). The primary health care unit (PHCU) comprises five satellite HPs (the lowest-level health facility at village level) and a referral HC. The secondary and tertiary level of care refers to the general hospitals and specialized hospitals respectively [13]. Both long acting and short acting contraceptive methods can be given at all levels of health care [14]. In addition, the government launched an implanon scale up program by task shifting; enabling health extension workers to insert implanon [15]. Family planning is also one of the packages of the health extension program, one of the flagship program in the Ethiopian health system [16].

The Ethiopia government planned to increase contraceptive prevalence rate to 55% in 2020. The government planned to increase implant and IUD to 33 and 15% respectively in the method mix [17]. All contraceptives including long acing and permanent contraceptive methods are provided free in Ethiopia [18]. Many governmental and nongovernmental organizations are providing long acting methods by outreach programs [14, 19]. Regardless of all these efforts, long acting and permanent contraceptive methods use is low. There is huge gap between total demand and demand satisfied. The current contraceptive method mix is dominated with short acting methods [7, 20]. Therefore, this study was designed to examine trends of long acting and permanent contraceptive methods use and identify factors associated with its use in Ethiopia.

## Methods

### Data

To identify factors associated with long acting and permanent contraceptive method use, the 2016 Ethiopian demographic and health survey (EDHS) data was used. The EDHS data was collected by the Central statistical Agency (CSA) at the request of the Federal Ministry of Health (FMoH). It was cross-sectional survey collected from January 18, 2016 to June 27, 2016.

The EDHS followed two stage stratified random sampling technique. Enumeration areas were selected in the first stage while households were selected in the second stage. The EDHS collected information about contraceptive use from all non-pregnant, fecund reproductive age women using structured and pretested questionnaire. From 15,683 reproductive age women interviewed for the 2016 EDHS, 9824 (62.6%) were married or in union at the time of survey. One thousand ninety women were excluded from the analysis because they reported being pregnant at the time of survey. Finally, 8734 married reproductive age women were included to identify factors associated with long acting and permanent contraceptive methods use. The data collectors were trained and had experience in data collection either in previous EDHS or other similar surveys. In addition, team supervisors, field editors, interviewers and secondary editors were recruited and trained by CSA. Data was collected and transferred to the CSA electronically via a secure Internet file streaming system (IFSS) and were stored on a password-protected computer. Then secondary editor resolves computer identified inconsistencies, code open ended questions and perform other activities. All four EDHS (EDHS 2000, 2005, 2011 and 2016) data were used to examine trends of long acting and permanent contraceptive method use.

#### Measurement

##### Outcome variable

The outcome variable for this study was contraceptive use. For this analysis, contraceptive use was grouped in to three categories; not using any method, using long acting and permanent contraceptive methods (IUD, female sterilization and implant) and using other methods (short acting and traditional).

##### Independent variables

The independent variables of the study were categorized in to three groups; socio-demographic, fertility and decision making related, and exposure to family planning programs. Some variables were recoded to have meaningful and small categories.

The main socio-demographic variables include;

- Age, region, place of residence, educational status, religion, occupation, working status and ethnicity of the woman, household wealth index, sex of head of the household, age, educational status and occupation of partner

Fertility and decision making related variables were;

- Fertility preference, desire for more children, ideal number of children, husband’s desire for more children, number of children ever born, age at first cohabitation, history of abortion
- Decision on how to spend earning, on health care, on large household purchase, to visit family and on first marriage

Family planning program exposure variables were

- Knowledge of the ovulatory period, knowledge of time of fertility, frequency of reading newspaper, frequency of listening radio, heard family planning messages on radio, heard family planning messages on TV, read family planning messages on newspaper, heard family planning messages by mobile phone, visited by health worker, health worker talked about family planning, visited health facility, told about family planning in the health facility.

##### Analytical methods

After getting permission, the data was downloaded from the DHS program official data base. Analysis was done using Stata 15.1. Open EPI software was used to examine whether there was significant linear trend in long acting and permanent contraceptive methods use in Ethiopia over time. Descriptive analysis was used to describe socio-economic and other variables of the study participants. Tables and graphs were used to present results. The data were weighted to consider the disproportionate sampling and non-response. In addition, the effect of sample design was handled when computing confidence intervals and standard errors. Before running the final model, multicollinearity was assessed using variance inflation factor. Variables highly correlated with other independent variables were excluded from the final multinomial logistic regression model. Permission to use data was obtained from the DHS programs.

## Result

### Socio-demographic characteristics of women

Majority of women were aged 20–34 years. Similarly, most of the participants (83.8%) were rural residents. About two third of mothers did not attend formal education. About 69 % of mothers were not working at the time of survey (Table 1).

**Table 1:** Socio-demographic characteristics of reproductive age married or in union women in Ethiopia, 2016

Variable	Frequency	Percent
Age		
15–19	501	5.5
20–24	1425	15.6
25–29	2075	22.7
30–34	1828	20.0
35–39	1487	16.3
40–44	1021	11.2
45–49	790	8.7
Place of residence		
Urban	1505	16.5
Rural	7622	83.5
Highest education attended		
No education	5666	62.1
Primary	2517	27.6
Secondary	563	6.2
Higher	381	4.2
Religion		
Orthodox	3793	41.6
Protestant	3066	33.6
Muslim	2050	22.5
Other	218	2.4
Wealth index		
Poorest	1708	18.7
Poorer	1814	19.9
Middle	1854	20.3
Richer	1806	19.8
Richest	1946	21.3
Husband/partner’s educational level		
No education	4203	46.1
Primary	3361	36.8
Secondary	846	9.3
Higher	643	7
Do not know	74	0.8
Husband’s/partner occupation		
Not working	729	8.0
Sales worker	623	6.8
Skilled agriculture	835	9.1
Subsistence farming	4604	50.4
Other	2336	25.6
Currently working		
Yes	6250	68.5
No	2877	31.5
Women’s occupation		
Not working	4664	51.1
Agriculture	2131	23.4
Sales	1225	13.4
Other	1106	12.1
Sex of head of the household		
Male	7941	87.0
Female	1186	13.0

### Fertility and decision making

The mean ages at first cohabitation and first sex were 17.2 (±4.0) and 16.8 (±3.3) years respectively. The mean number of children ever born was 3.8 (±2.8). The survey indicated that women in Ethiopia had limited knowledge about fertile period. Among all married reproductive age women, only 22.9% correctly know the ovulatory period and only 56.8% knew a woman can get pregnant after birth and before period. A little more than half of the mothers desired to have other children. Majority of women cohabited at age less than 20 years. About two third (60.7%) women reported that their marriage was arranged by parents. About 66 % of women reported that their health care was decided jointly (by partner and themselves) (Table 2).

**Table 2:** Fertility and decision making characteristics of married or in union reproductive age women in Ethiopia, 2016

Variable	Frequency	Percent
Knowledge of ovulatory period		
During her period	352	3.9
After period ended	2518	27.6
Middle of the cycle	2093	22.9
Before period begins	661	7.2
At any time	1894	20.7
Do not know	1610	17.6
History of abortion		
No	8148	89.3
Yes	979	10.7
Pregnancy can occur after birth and before period		
No	3349	36.7
Yes	5183	56.8
Do not know	595	6.5
Fertility preference		
Have another	5084	55.7
Undecided	460	5.0
No more	3376	37.0
Other	206	2.3
Ideal number of children		
No child	720	7.9
1–5	4327	47.4
> 5	2926	32.1
Non numeric	1155	12.7
Concordance on number of children		
Both wants same	3579	39.4
Husband wants more	2341	25.8
Husband wants fewer	662	7.3
Do not know	2503	27.6
Number of children ever born		
No child	667	7.3
1–4	4688	51.4
> 4	3771	41.3
Number of living children		
No living child	709	7.8
1–3 children	4031	44.2
4 or more children	4387	48.1
Age at first cohabitation		
≤ 19 years	7224	79.1
20–24 years	1476	16.2
25–29	330	3.6
30 or more	97	1.1
Decision maker on the respondent’s health care		
Respondent alone	1434	15.7
Respondent & partner	6029	66.1
Partner alone	1623	17.8
Other	41	0.4
Decision maker on large household purchase		
Respondent alone		
Respondent & partner	993	10.9
Partner alone	6181	67.7
Other	1912	20.9
	40	0.4
Decision maker to visit family or relatives		
Respondent alone	1670	18.3
Respondent & partner	6007	65.8
Partner alone	1422	15.6
Other	28	0.3
Decision maker on what to do with husband money		
Respondent alone	636	7.0
Respondent & partner	6291	68.9
Partner alone	1998	21.9
Other	148	1.6
Decision maker on respondent’s first marriage		
Self	3215	35.2
Parents	5536	60.7
Other	376	4.1

### Exposure to family planning programs

Majority of women did not read newspaper at all (91.7%) did not listen radio at all (69.7%) and did not watch TV at all (76.7%). About 79% women did not own mobile phone. Almost all (97.6%) women never used internet. Only 22.2, 14.1 and 3.3% women reported that they had heard family planning messages on radio, watched on TV and read on newspaper/magazine respectively on the last few months. Only 2.1% women reported that they had received family planning related text message on mobile on the last few months. Many women (70.3%) reported that they were not visited by health worker in the last 12 months (Table 3).

**Table 3:** Exposure to mass media and family planning messages among married or in union reproductive age women in Ethiopia, 2016

Exposure variable	Number	Percent
Frequency of reading newspaper		
Not at all	8371	91.7
Less than once a week	552	6.0
At least once a week	205	2.2
Frequency of listening radio		
Not at all	6360	69.7
Less than once a week	1383	15.2
At least once a week	1384	15.2
Frequency of watching TV		
Not at all	6996	76.7
Less than once a week	1013	11.1
At least once a week	1117	12.2
Own mobile phone		
No	7192	78.8
Yes	1935	21.2
Use of internet		
Never	8911	97.6
Yes	216	2.3
Heard family planning message on radio on last few months		
No	7103	77.8
Yes	2024	22.2
Heard family planning messages on TV on last few months		
No	7837	85.9
Yes	1290	14.1
Read about family planning messages on newspaper/magazine last few months		
No	8827	96.7
Yes	300	3.3
Received family planning text message on mobile phone		
No	8936	97.9
Yes	191	2.1
Visited by field worker in the last 12 months		
No	6431	70.5
Yes	2696	29.5
Field worker talk about family planning		
No	1068	39.6
Yes	1628	60.4
Visited health facility in the last 12 months		
No	4682	51.3
Yes	4445	48.7
Told about family planning in the health facility		
No	2669	60.0
Yes	1777	40.0

### Contraceptive use

About 59 % (95%CI: 55.9–63.0%) women reported that they had ever used or tried something to delay or avoid pregnancy. About one-fourth of mothers (95%CI 26.4–30.9%) reported that they were using short acting or traditional contraceptives methods while 11.6% (95%CI 10.2–13.1%) were using long acting or permanent contraceptive methods. Specifically, 8.8% (95%CI: 7.7–10.1%) and 2.3% (95%CI: 1.7–3.0%) women were using implants and IUD respectively.

### Trends in LAPM contraceptive method use

**Fig. 1:**
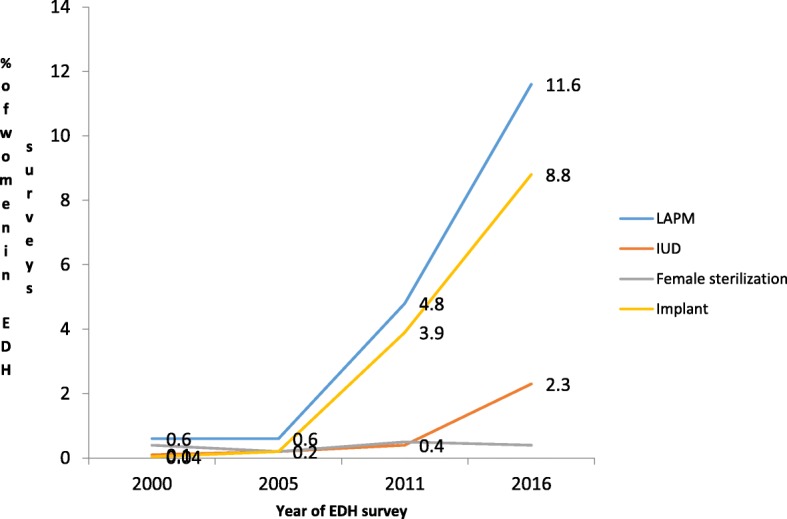
Trends in long acting and permanent contraceptive method use in Ethiopia, from 2000 to 2016

**Fig. 2:**
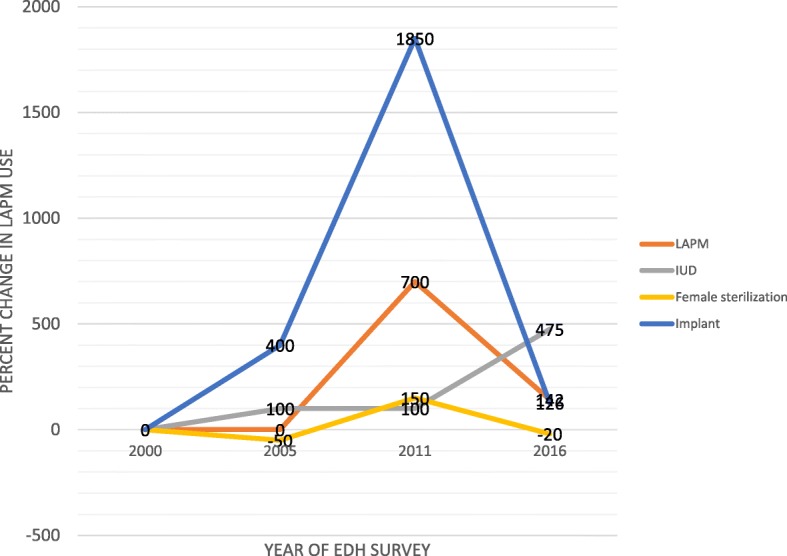
Percent change in long acting and permanent contraceptive method use among married or in union reproductive age women in Ethiopia, 2000–2016

Long acting and permanent contraceptive method use increased significantly (Extended MH chi square for linear trend = 1421.15, *p* < 0.01) from 0.5% in 2000 to 11.6% in 2016. Implant use showed the highest change from 0.04% in 2000 to 8.8% in 2016 (Extended MH chi square for linear trend = 1231.41, p < 0.01) (Fig. 1). When analyzing the percent change in long acting and permanent contraceptive method use, the highest percentage of change was observed between 2005 and 2011. Both female sterilization and implant use showed the highest percentage increase at this period. But for IUD use, the highest increase was observed from 2011 to 2016 (Fig. 2).

### Factors associated with long acting and permanent contraceptive method use

Multinomial logistic regression model was fitted to identify factors associated with long acting and permanent contraceptive method use. The analysis indicated that wealth index, occupation, sex of head of the household, history of abortion and ideal number of children were significantly associated with long acting and permanent contraceptive methods use (Table 4).

**Table 4:** Factors associated with long acting and permanent contraceptive methods use in Ethiopia, 2016

Variable	LAPM use AOR(95%CI)
Region	
Tigray	1.0
Amhara	1.3(0.4–4.4)
Oromia	0.4(0.1–1.7)
SNNPR	1.1(0.2–5.6)
Addis Ababa	1.5(0.5–5.2)
Other regions	0.4(0.1–1.4)
Highest education attended	
No education	1.0
Primary	0.9(0.5–1.5)
Secondary	0.8(0.3–1.9)
Higher	1.4(0.5–3.7)
Religion	
Orthodox	1.0
Muslim	0.9(0.4–2.0)
Protestant	2.0(0.8–4.6)
Other	0.03(0.005–0.2)
Ethnicity	
Amhara	1.0
Oromo	1.4 (0.5–4.0)
Tigrie	0.9(0.3–2.9)
Sidama	0.7(0.1–3.4)
Welaita	0. 3(0.04–2.8)
Other	0.4(0.1–1.2)
Wealth index	
Poorest	1.00
Poorer	1.5(0.7–3.3)
Middle	1.8(0.9–3.8)
Richer	2.6(1.2–5.4)**
Richest	2.1(0.8–7.3)
Husband’s/partner occupation	
Did not work	1.0
Sales worker	2.0(0.6–6.5)
Skilled agriculture	2.2(0.7–7.3)
Subsistence farming	2.2(0.7–7.3)
Other	2.4(0.8–7.3)
Women’s occupation	
Not working	1.0
Agriculture	1.7(0.9–2.9)
Sales	2.1(1.1–3.9)*
Other	1.7(1.0–3.1)
Sex of head of the household	
Female	0.2(0.1–0.5)***
Male	1.00
Use of internet	
Never	1.0
Yes	1.6(0.7–4.1)
Time of ovulation	
Middle of the cycle	1.0
Other	0.8(0.5–1.3)
History of abortion	
No	1.0
Yes	0.2 (0.1–0.5)***
Pregnancy can occur after birth and before period	
No	1.0
Yes	1.0(0.7–1.5)
Ideal number of children	
No child	1.0
1–5 children	4.2(1.4–13.0)*
More than five	2.1(0.7–6.1)
Non numeric	2.2(0.6–7.8)
Concordance on number of children	
Husband wants more	1.0
Both wants same	0.7(0.4–1.1)
Husband wants fewer	0.7(0.4–1.1)
Do not know	0.6(0.3–1.0)
Number of living children	1.00(0.8–1.1)
Age at first cohabitation	1.0(0.8–1.0)
Heard family planning on radio on last few months	
No	1.0
Yes	0.9(0.7–1.3)
Hear family planning by text message on mobile phone	
No	1.0
Yes	2.6(0.8–8.7)
Heard about family planning at community event/conversation	
No	1.0
Yes	0.7(0.5–1.1)
Field worker talked about family planning	
No	1.0
Yes	1.4(0.9–2.1)
Visited health facility in the last 12 months	
No	1.0
Yes	0.8(0.5–1.3)
Decision on your first marriage	
Self	1.0
Parents	0.7(0.4–1.1)
Other	1.0(0.4–1.5)

The odds of using long acting and permanent contraceptive methods compared to those not using any method for women in the richer wealth index was 2.6 (AOR 2.6; 95%CI 1.2–5.4) times higher compared to women in the poorest wealth quintile. Similarly, the odds of using long acting and permanent contraceptive methods compared to not using for women who were sales worker was 2.1 (AOR 2.1; 95%CI 1.1–3.9) times higher compared to women who were not working at the time of survey.

The odds of using long acting and permanent contraceptive methods compared to not using for women living in female headed households was 80% lower (AOR 0.2; 95%CI 0.1–0.5) compared to women living in male headed household. The odds of using long acting and permanent of contraceptive methods compared to not using for women who had history of abortion was 80% lower (AOR 0.5; 95%CI 0.1–0.5) compared to women who had no history of abortion. Similarly, The odds of using long acting and permanent method of contraceptive compared to not using for women whose ideal number of children is 1–5 was 4.2 (AOR; 4.2, 95%CI 1.4–13.0) times higher compared to women who desired no more children.

## Discussion

### Long acting and permanent contraceptive method use

This analysis identified that only 11.6% Ethiopian mothers were using long acting or permanent contraceptive methods. About 9 and 2% of mothers were using implants and IUD respectively. The proportion of women using female sterilization (0.4%) was almost negligible. Vasectomy was nil. The proportion of women using long acting and permanent contraceptives methods was very low compared to the national family planning target of 2020 and the existing high demand for long acting and permanent contraceptive methods. The current practice was also low compared to the national health sector transformation plan and the family planning coasted implementation targets (to increase contraceptive prevalence rate to 55% at the end of 2020 by increasing the share of implant, IUD, female sterilization and vasectomy to 33, 15, 1.5 and 0.5% respectively in the method mix) [20]. In addition, the method mix was dominated by short acting contraceptive methods. Therefore, the government shall intensify task shifting activities launched on 2009. Awareness creation activities to avoid the prevailing myths and misconceptions are crucial.

The proportion of women using long acting and permanent contraceptive methods in this study (11.6%) was much lower than the level of use at global level (which accounted for 56%). In 2015, 19% of married or in-union women relied on female sterilization and 14% used the IUD [7]. The proportion of women using long acting and permanent contraceptive methods in Ethiopia was lower than a study conducted in Kampala, Uganda [21]. But it was similar with a study conducted in Rwanda (10.4%) [22].

The level of long acting and permanent contraceptive method use in this study was lower than meta-analysis done in Ethiopia [23]. The reason for this is that most of the studies included in the meta-analysis were facility based and based on urban settings both of which increase the proportion of women using long acting and permanent contraceptive methods [24–29].

### Trends of long acting and permanent contraceptive method use

Long acting and permanent contraceptive methods use increased significantly (Extended MH Chi square for linear trend = 1421.15, *p* < 0.01) in the 16 years period although the level of use was still low compared to the national targets and the existing demand. This finding was similar with a study conducted in Lusaka Zambia which showed that long acting and reversible method use increased from less than 1 to 9% from 2004 to 2011 [30].

Implant use showed the highest change from 0.04% use in 2000 to 8.8% in 2016. This may be due to the task shift designed by the ministry of health. Health extension workers were trained to insert implants [31]. This made the implant accessible to the rural women. The highest percentage change in long acting and permanent contraceptive methods use was observed between 2005 and 2011. The percent change in long acting and permanent contraceptive use was lower from 2011 to 2016. The reason for this may be the presence of high demand for long acting and permanent contraceptive methods from 2005 to 2011. In addition, the government exerted significant effort to address that need at that time. Since that huge gap was addressed from 20,005 to 2011, the percent change from 2011 to 2016 seems lower. But this does not mean long acting and permanent contraceptive method use was lower. Both female sterilization and implant showed the highest percentage increase at this period. But for IUD use, the highest increase was observed from 2011 to 2016. Further qualitative study is needed to explore the reason and learn to increase long acting and permanent contraceptive methods use in Ethiopia.

### Factors associated with long acting and permanent contraceptive method use

The odds of using long acting and permanent contraceptive methods compared to non-use for women in the richer wealth index was higher compared to women in the poorest wealth quintile. This finding was similar with a multi country study conducted in developing countries based on DHS data and the 2011 EDHS data [32, 33]. This may be due to financial barriers to access long acting and permanent methods of contraceptives in Ethiopia. Except implants which are available in health posts, other long acting and permanent contraceptive methods are not easily accessible in Ethiopia. The users have to travel to access these methods of contraceptives. In addition, there is belief or misconception that long acting and permanent contraceptive methods are not convenient for working women [11, 34]. And the poorest are more likely to engage in activities which are more labor intensive. Because of this, the poor may refrain from using these methods.

The odds of using long acting and permanent contraceptive methods compared to non-use for women who were sales worker was higher compared to women who were not working. Many studies in Ethiopia identified that occupation is associated with long acting and permanent contraceptive methods use. A study in Harar city indicated that daily laborers were less likely to use long acting reversible contraceptive methods compared to house wives [35]. A study conducted in Jigjiga showed that employee women were more likely to use long acting and permanent contraceptive methods compared to house wives [36]. Similar study in Areka town, Southern Ethiopia, indicated that government employees were more likely to use long acting and reversible methods [37]. The possible reason for this is that sales workers are better educated than others which improves their information processing skill. From the data, about 33% of nonworking women attended no education but only 6.2% of sales women did so. Sales workers had also better access to mass media which exposes them to family planning messages. There are evidences in Ethiopia that indicate educated women are more likely to use long acting and permanent contraceptive methods compared to women who did not attend formal education [35, 38, 39].

The odds of using long acting and permanent contraceptive methods compared to non-use for women living in female headed households was lower compared to women living in male headed household. This finding was similar with a study conducted in Lesotho [40]. The possible reason for this may be that female headed households do have less frequent sexual intercourse compared to male headed households. The husband may be away from home for different reasons. As a result the woman may not use contraceptives or may use short acting methods.

The odds of using long acting and permanent contraceptive methods compared to non-use for women who had history of abortion was lower compared to women who had no history of abortion. This finding contradicts a study in Luanda, Angola, which indicated that history of abortion was associated with contraceptive use [41]. The reason for this may be that women with abortion may have desire for other children. These women may not use contraceptives or will use short acting methods. Women who had abortion may also associate the abortion with long acting methods use and refrain from using.

The odds of using long acting and permanent methods of contraceptive compared to not using for women whose ideal number of children is 1–5 was higher compared to women who did not want any child. This finding contradict with a community based study conducted in Amhara region, Ethiopia which indicated that women with higher ideal number of children are less likely to use long acting methods [42]. The reason for this may be that women who desired no children are older women who are using short acting methods or not using any method thinking that they are approaching menopause.

The strength of this study is that it is based on nationally representative, large data. On the other hand, some factors are not included in the analysis as it is secondary data.

## Conclusion

Although increasing significantly, the current level of long acting and permanent contraceptive methods use was still low in Ethiopia compared to the national targets. The odds of long acting and permanent contraceptive methods use were high among women in the richer wealth index, among women who were sales workers and among women who desired more children. On the other hand, the odds of use were lower among women with female headed households and women with history of abortion.

Further research is needed to identify the different levels of change in long acting and contraceptive use in Ethiopia. Emphasis should be given to women in the lower wealth quintile and women who had history of abortion to improve long acting and permanent contraceptive method use.

## Data Availability

The data sets analyzed during this study are available in the DHS programs repository available at https://dhsprogram.com/

## Abbreviations

AOR: Adjusted Odds Ratio
CSA: Central Statistical Agency
EDHS: Ethiopian Demographic and Health Survey
FMoH: Federal Ministry of Health
IUD: Intrauterine Device
LAPM: Long Acting and Permanent contraceptive method
MH: Mantel-Haenszel
SSA: Sub-Saharan Africa
TV: Television
